# Effect of Mill-Rejected Granular Cement Grains on Healing Concrete Cracks

**DOI:** 10.3390/ma13040840

**Published:** 2020-02-12

**Authors:** Feseha Sahile Asrat, Tewodros Tekeste Ghebrab

**Affiliations:** 1 Civil Engineering, Jimma University Institute of Technology, P.O. Box 378, Jimma, Ethiopia; 2 Civil, Environmental, and Construction Engineering, Texas Tech University, Lubbock, TX 79409-1023, USA; Tewodros.ghebrab@ttu.edu

**Keywords:** MRGC, artificial crack, crack healing, strength regain, water permeability

## Abstract

The effect of mill-rejected granular cement (MRGC) on enabling concrete to autogenously heal its cracks was investigated. The crack-healing efficiency of concrete containing 5%, 10%, 15%, and 20% wt. of MRGC as a replacement for natural fine aggregate was investigated at the age of 28 days. Concrete specimens were induced with artificial cracks and placed in water or air at 20 ± 2 °C to cure and heal the cracks for an additional 28 days. Compressive, flexural, and tensile strengths and water permeability tests were carried out to evaluate crack-healing by evaluating the strength to regain and the reduction in water permeability of concrete. For the air-cured specimens, the gain in compressive strength was between 45% and 79%, the flexural strength was between 74% and 87%, and the tensile strength was between 75% and 84% of the reference specimens for the MRGC content was between 0% and 20%, respectively. For the water-cured specimens, the gain in compressive strength was between 54% and 92%, the flexural strength was between 76% and 94%, the tensile strength was between 83% and 96% of the reference specimens for the MRGC content between 0% and 20%. The water permeability coefficients of the concrete specimens cured in water after cracking decreased by one order of magnitude, while those of the specimens cured in the air increased by the same order of magnitude. The crack-healing efficiency of concrete could be enhanced by increasing the MRGC content of concrete and hydration water.

## 1. Introduction 

Cement concrete is a man-made composite rock composed of coarse aggregate, fine aggregate, and cement paste. The conventional cement concrete referred to as concrete uses ordinary Portland cement (OPC) paste to bind the aggregates and fill the voids between them. Therefore, cement paste plays a vital role in the properties and performance of concrete. When OPC is mixed with water, a chemical reaction (hydration) takes place and gives off various products. Calcium silicate hydrate (C-S-H) is a predominant cement hydration product responsible for the cementitious property of cement paste. Generally, the strength of concrete could be defined as the bond strength between the C-S-H gel and the aggregates.

Concrete is the most common construction material worldwide. According to Meyers [1], the global annual production of concrete had exceeded 10 billion tons in the last decade. This figure is expected to grow with the increasing demand for concrete in the construction sectors. Concrete is commonly used for the construction of several infrastructures such as buildings, roads, and dams. The qualities that make concrete popular construction material are its versatility, durability, and cost-effectiveness, and that it can be produced from locally available raw materials. 

Concrete, even though it has many advantages, has some limitations that negatively affect its performance. For example, the fact that concrete being strong in compression but weak in tension makes concrete more susceptible to cracking under minimal stress intensities. Concrete cracks form when the tensile stress exceeds the tensile strength of the concrete. The tensile stress in concrete can be induced by internal or external factors such as shrinkage, expansive chemical reactions, temperature variation, freeze-thaw cycles, poor construction practice, external loads, etc. [2,3]. Crack formation in concrete could affect the strength, durability, serviceability, and aesthetics of a concrete structure. If concrete cracks are not treated properly, they may lead to a complete failure of the structure [4,5,6] by allowing the ingression of chemical solutions detrimental to concrete. Some of the common chemical attacks on concrete are delayed ettringite formation due to the ingression of excess sulfate, corrosion of reinforcement due to the ingression of chloride ions, and an alkali-silicate reaction. Therefore, to improve concrete durability, the ingression of moisture and aggressive chemicals into concrete should be prevented by sealing the cracks.

Several researchers have introduced some techniques for the maintenance and repair of cracks in concrete structures [7,8,9,10]. Some of these techniques allow concrete to heal the cracks by itself without any human intervention [11,12]. Self-healing mechanisms in concrete are categorized as autonomous and autogenous. Autonomous self-healing concrete is designed to heal its cracks using special additives such as bacteria in bioremediation techniques [13,14,15,16,17,18,19,20], fiber in fiber-reinforced strain-hardening cementitious composites [10,21,22,23,24], supplementary cementitious materials (fly ash, silica fume, blast-furnace slag, etc.) [25], geo-materials in the form of powder, and an expansive agent, which expands and fills the crack void [26,27]. Autogenous self-healing concrete (natural or intrinsic self-healing) is designed to heal its cracks throughout its service life without any special additives [28]. An autogenous self-healing mechanism involves the filling of crack space with calcium carbonate (CaCO_3_), C-S-H, crystallization of calcium hydroxide (Ca(OH)_2_, and sediments. When water containing dissolved atmospheric carbon dioxide enters concrete cracks, the carbonation reaction takes place between the carbon dioxide and calcium hydroxide present near the cracked surfaces. CaCO_3_ produced from the carbonation reaction may fill the crack space to prevent further ingression of water. The crystallization of newly formed calcium hydroxide on the cracked surfaces could also play a role in narrowing down the crack widths by filling up the crack space. Crack space can also be filled with sediments present in the water entering the cracks. Unhydrated cement particles present in the vicinity of the cracked surfaces’ hydrate in the water entering the cracks produce C-S-H gel, which could also fill the crack space and bind the cracked surfaces [8,29,30,31].

One of the autogenous self-healing mechanisms discussed above is the crack filling and cracked surface binding effect of the hydration of the unhydrated cement particles. However, the effectiveness of this mechanism depends on the amount of the unhydrated cement particles present during crack formation. The amount of unhydrated cement particles present in cement paste depends on several factors such as moisture, curing mechanism, age, etc. [2]. However, no report on the use of unhydrated cement particles as autogenous self-healing agents was found in the literature. 

This paper presents the work done to investigate the use of coarse cement grains as crack-healing agents in concrete. Coarse cement grains, classified as mill-rejected granular cement (MRGC), were obtained from cement industries. MRGC is coarse cement that did not meet the required fineness and did not separate to be reprocessed. The main objective of this research work is to evaluate the effect of MRGC and its amount on the self-healing performance of concrete. Concrete mixes containing different amounts of MRGC as a partial replacement for fine aggregate were tested. Artificial cracks were induced on the specimens and allowed to heal the cracks. The crack-healing performance of the concrete specimens was evaluated based on the percentage regain in strength and impermeability after the specimens were allowed to heal the induced cracks.

## 2. Materials and Methods

### 2.1. Materials

Concrete specimens were prepared using locally available materials with the partial replacement of MRGC for a natural fine aggregate. The materials used in this study are presented below. 

#### 2.1.1. Cement

Ordinary Portland cement (OPC) obtained from Ethio Cement Factory (Chancho, Oromiya, Ethiopia) was used in this study. The OPC used in this study complies with CEM I Portland cement as in BS EN 197-1:2011 [32]. The physical properties and chemical composition of the cement are shown in Table 1.

#### 2.1.2. Mill-Rejected Granular Cement (MRGC)

**Figure 1:**
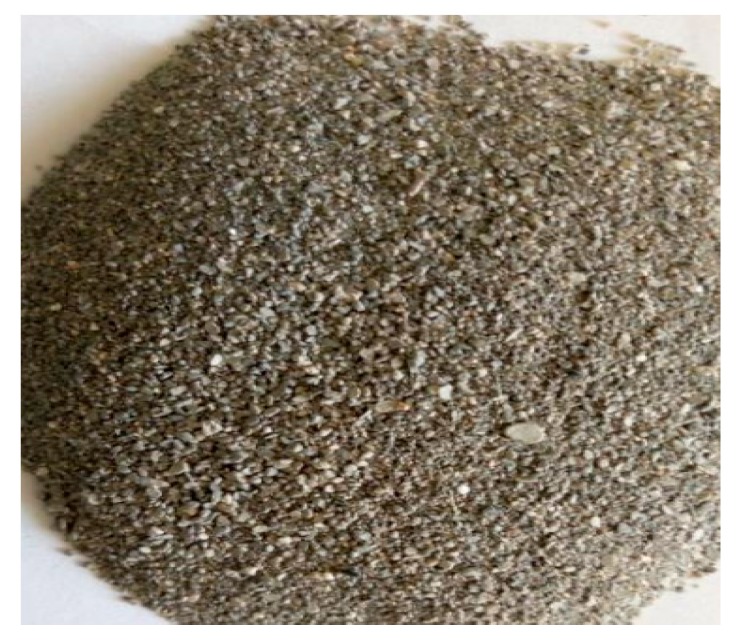
MRCG retained in sieve No. 200.

Mill-rejected granular cement (MRGC) was also obtained from the same source asthe OPC. MRGC, as the name indicates, is a portion of cement rejected by the cement processing because it has a grain size coarser than the specified 75 μm sieve size. The chemical compositions of the MRGC was the same as that of OPC (Table 1). The grain size of the MRGC used in this study was between 0.075 mm and 1.18 mm. A sample of the MRGC used is shown in Figure 1.

**Figure 2:**
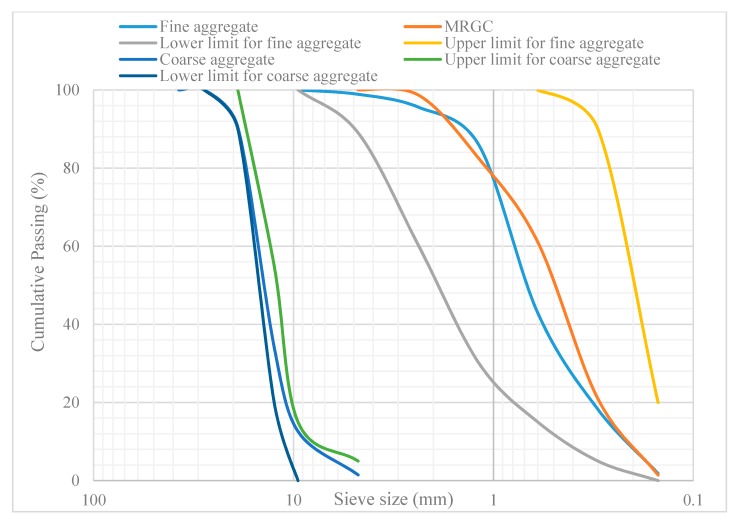
Grain size distribution of MRGC, fine aggregate, coarse aggregate, and standard limits.

Grain size analysis was performed on the MRGC according to the BS EN 933-1 [33], which is a standard procedure for sieve analysis of fine aggregates. The resulting grain size distribution curve is shown in Figure 2. The fineness modulus of the MRGC was 2.36. From the physical property test results and particle size distributions shown in Figure 2, the MRGC satisfied the standard requirements for use in a concrete mix.

#### 2.1.3. Fine Aggregate

Natural sand with 4.75 mm in maximum size, obtained from Gibe riverbed located about 180 km southwest of Addis Ababa, Ethiopia, was used for concrete mix. Grainsize analysis of the sand was performed, according to the BS EN 933-1 standard procedure [33]. The fineness modulus, unit weight (per BS EN 1097-3 [34]), specific gravity (per BS EN 1097-6 [35]), and water absorption (per BS EN 1097-6 [36]) were2.66, 1630 kg/m^3^,2.70, and 1.26%, respectively.

#### 2.1.4. Coarse Aggregate

Crushed stone with a 20-mm maximum size, obtained from a quarry on the outskirts of Addis Ababa, Ethiopia, was used for concrete mix. Grain size analysis of the coarse aggregate was performed in accordance with BS EN 933-1standard procedure [33]. The specific gravity and water absorption capacity of the aggregate were 2.74% and 1.30%, respectively, determined using BS EN 1097-6 [35]. Abrasion value of 26.8% was obtained using BS EN 1097-8 test procedure [36].

The physical properties and grain size distribution (Figure 2) indicate that the fine and coarse aggregates met the specifications for concrete mix [37,38].

### 2.2. Experimental Methods

The presented study was carried out by preparing concrete mixes containing different percentages of MRGC as a partial replacement for sand. At the age of 28 days, the specimens were cracked using controlled compressive, tensile, or flexural stresses. When cracks visible to the naked eye started to appear, the loading stopped. The stress levels causing the initial visible cracks were recorded. Some of the cracked specimens were placed in air at room temperature and others in water at room temperature for 28 days to heal their cracks. After 28 days, the specimens were tested to determine the regain in compression, tensile, and flexural strengths. The change in permeability after crack-healing was also used to evaluate the crack-healing efficiency of concrete containing MRGC.

Crack healing in the concrete was assessed based on the evaluation of the regain in the compressive, tensile, and flexural strengths and a drop in permeability values obtained when the specimens were tested after the crack healing period. The results obtained after the healing period were compared with the performance of the uncracked reference specimens to determine the percentage regain in strength or drop in permeability due to crack healing.

#### 2.2.1. Concrete Mixing and Specimen Details

The concrete mix was designed with a targeted compressive strength of 25 MPa in accordance with BS EN 206-1 [37] and BS 8500-2 [38] standard procedures. A water-to-cement ratio (w/c) of 0.47 was used in the mix. This value was selected based on the evaluation of the performance of MRCG on the mechanical and physical properties of concrete. Furthermore, the same w/c ratio was used in all mixes to avoid the influence of the w/c ratio on the crack-healing mechanism of MRGC. Five concrete mixes were prepared by varying the percentage replacement of MRGC for sand. The control mix (C0) was prepared using sand only, while other mixes were prepared by replacing sand with 5%, 10%, 15%, and 20% by weight of MRCG, as shown in Table 2. The designation of each mix was given such that the number following letters C represents the weight percentage replacement of MRGC for natural sand.

The concrete mix was performed by adding half of the coarse aggregate, sand, MRGC, and the remaining half of the coarse aggregate, consecutively, into a 300-L capacity tilt drum mixer. After the mixer was run for 30 s, about half of the water was added within the next 15 s. The mixer was run for a total of 3 min and stopped for 10 min. After adding the cement and run the mixer for 30 s, the remaining water was added within the next 30 s and the mixing was continued for 2 min afterward. The mixing and sampling of fresh concrete were carried out in accordance with the BS 1881-125 [39] standard procedure.

The slump test was carried out on each concrete mix in accordance with BS EN 12350-2 [40]. The slump test results obtained for each concrete mix are presented in Table 3.

Concrete specimens were cast and compacted using a vibration table to ensure good compaction. The surface of each specimen was leveled with a trowel and sealed off with a plastic cover to avoid moisture loss. After the specimens were air-dried at a temperature of 20 ± 3 °C for 24 h, they were placed in a water bath with a temperature of 20 ± 2 °C for 28 days. During the testing times, the specimens were removed from the water bath and wiped off with a damp cloth to remove any excess water from the surface.

Concrete specimens prepared for each type of test were 100 mm × 100 mm × 500 mm prisms for a four-point bending test, 150-mm cubes for a compressive strength test, 100-mm diameter and 200-mm long cylinders for an indirect tensile strength test, and 200-mm diameter and 100-mm thick discs for a water permeability test. Three specimens were prepared for each concrete mix shown in Table 2.

#### 2.2.2. Artificial Cracking

To investigate the effect of MRCG and its percentage replacement of the natural sand on enabling concrete to autogenously heal its cracks, artificial cracks were introduced into the specimens. At the age of 28 days, fine cracks were induced into the specimens using displacement controlled compressive stress, split tensile stress, and flexural stress. The loading was stopped when cracks visible to the naked eye started to emerge, and the cracking load and the corresponding displacement were recorded. The cracked specimens were unloaded, placed in air or water at room temperature to heal the cracks, and tested 28 days after the initial cracking.

#### 2.2.3. Crack Formation Using Compressive Stress

The concrete cube specimens were compressed under a displacement control mode using a standard compression machine. The cubes were loaded until cracks started to appear on the surface of the specimen. The loading of the specimens was stopped when the cracks appeared, and the final displacement was taken as a control. The crack-inducing compressive stress was determined by dividing the cracking load by the cross-sectional area of the specimen. Six replicates of concrete specimens were cracked for each concrete mix, and three of them were immersed in a water bath while the remaining three were placed outside to cure in the air until they were tested.

#### 2.2.4. Crack Formation Using Split Tensile Stress

To create artificial cracks using the indirect tensile stress, the cylindrical specimens were loaded diametrically across the circular cross-section until cracking occurred. The loading stopped after the formation of the cracks, and the peak load corresponding to the crack formation was recorded along with the displacement of the load.

#### 2.2.5. Crack Formation Using Flexural Stress

**Figure 3:**
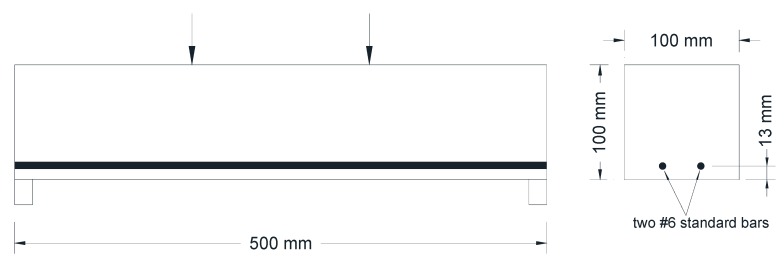
Schematic of the four-point bending test setup and beam reinforcement.

Four-point bending was another method used to create artificial cracks in the concrete specimens. The prismatic specimens were placed on two parallel supports, and the load was applied on them through two parallel pins placed at the third points of the specimens. The bending load was applied to the specimens until the first visible flexural crack forms. To prevent total collapse and to control the crack width, the beams were reinforced with two #6 standard reinforcing bars, as shown in Figure 3. The reinforcement of the specimens was beneficial in controlling crack width, enabling the formation of distributed cracks along the beam length, and preventing the development of a single crack opening at the midspan. The maximum deflection of the specimen during the formation of the cracks was recorded to be used as a control for the reloading during the post crack healing test.

#### 2.2.6. Evaluation of the Crack-Healing Effect of MRGC

The effect of MRCG on healing concrete cracks was evaluated based on strength and permeability test results. The specimens induced with artificial cracks, after being submerged in water at a temperature of 20 ± 3 °C for a specified period, were tested to investigate if there was any strength to regain in the specimens due to autogenous healing of the cracks. The crack healing efficiency of MRGC was evaluated by comparing the initial cracking strength and the reloading strength after crack-healing as percentages of the 28-day strength [6]. Compressive strength test, indirect tensile strength test, and flexural strength test were used to evaluate the strength regained. Furthermore, a permeability test was carried out to investigate the crack healing effect of MRGC.

#### 2.2.7. Compressive Strength Test

Compressive strength tests were carried out on the initially cracked and water-cured and air-cured cubic specimens 28 days after cracking. The test was performed in accordance with BS EN 12390-3:2019 [41] standard procedures. The specimens were loaded at a rate of 0.5 MPa/s until they fail, and the average peak compressive stress at failure was recorded as the post-self-healing compressive strength. The regain in strength due to autogenous crack healing of the water-cured specimens at the level of initial cracking was compared with those of the air-dried specimens as a percentage of the 28-day compressive strength.

#### 2.2.8. Indirect Tensile Strength Test

Splitting tensile strength tests were carried out on the initially cracked and water-cured and air-cured cubic specimens 28 days after cracking. The test was performed in accordance with BS EN 12390-6:2019 [42] standard procedures. The specimens were subjected to a splitting load at a constant rate of 1300 N/s until they fail. Regaining tensile strength due to autogenous crack healing of the water-cured specimens at the level of initial cracking was compared with those of the air-dried specimens as a percentage of the 28-day tensile strength.

#### 2.2.9. Flexural Strength Test

Four-point bending strength tests were carried out on the initially cracked and water-cured and air-dried cubic specimens 28 days after cracking. The test was performed in accordance with BS EN 12390-5:2019 [43] standard testing procedures. The specimens were subjected to a four-point bending load at a standard rate until they fail. The regain in flexural strength due to autogenous crack healing of the water-cured specimens at the level of initial cracking was compared with those of the air-dried specimens as a percentage of the 28-day flexural strength.

#### 2.2.10. Water Permeability Test

Water permeability of concrete was also one of the methods used to evaluate the effect of MRGC on enabling concrete to autogenously heal its cracks. The crack healing efficiency was evaluated by comparing the permeability values of the specimens measured before cracking, after cracking, and the crack-healing period. After the specimens were cracked at 28 days, they were cured in the air or in water until the testing day of 28 days. The constant head permeability test was carried out on concrete discs that are 200 mm in diameter and 100 mm in thickness using the UTC 1080 apparatus, following a BS EN 12390-8 [44] standard procedure. The circumference of the saturated surface dry cylindrical concrete specimens was sealed with a watertight plastic before they were placed into a permeameter cell filled with de-aired water at an ambient temperature of 23°C. The inflow side of the specimens was subjected to an 800 kPa constant pressure head for 48 h. The constant pressure was maintained by connecting an air compressor to a water tank, and pressure regulators controlled the pressure. The volumetric inflow and outflow of the water and the ambient temperature were monitored. When a steady-state flow rate was achieved, the volume flow was plotted against time until the slope of the inflow and outflow line was achieved. The mean of the inflow and outflow rate plots was used to calculate the permeability. Two replicates of the specimen were tested for each concrete mix, and the average values were considered.

The coefficient of permeability *k* (m/s) was calculated using the following equation.

(1) $$k\;=\;\frac{VL}{tAh}$$

where k = D’Arcy Coefficient of Permeability (m/s), *V* = volume of water (m^3^), *L*= length of the concrete specimen (m), *t* = elapsed time (s), *A* = area of the specimen (m^2^), and *h* = applied pressure head (m).

## 3. Results and Discussions

### 3.1. Slump Test Results

The results indicate that the workability of concrete decreases with the increase in MRGC content. This is in agreement with the fact that the workability of concrete decreases with an increase in the content of a fine aggregate. Therefore, the amount of mixing water or a superplasticizer should be increased with an increase in the MRGC content of concrete to improve its workability.

### 3.2. Strength Regains

#### 3.2.1. Compressive Strength

**Figure 4:**
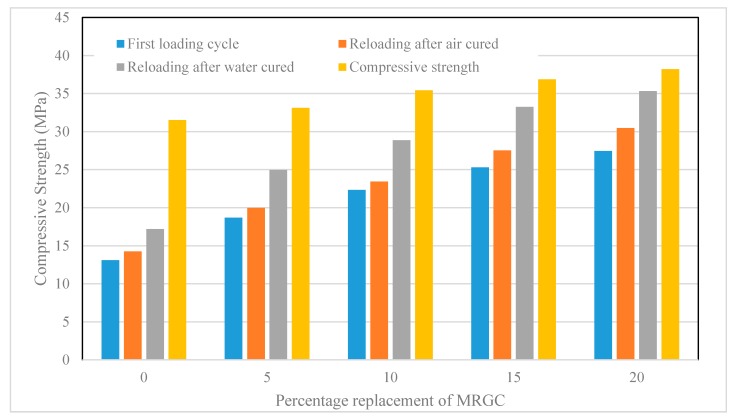
Compressive strength regained due to a crack healing effect of MRGC.

The first cycle compressive strength, which is the compressive stress required to induce cracks, and the compressive strength regained due to crack healing after the specimens were cured in air and in water are presented in Table 4. A graphical demonstration of the test results is shown in Figure 4.

It was observed that the percentage regained in compressive strength by the control mix (C0) after the crack-healing period was 45.23% and 54.53% for the air-cured and water-cured specimens, respectively. The percentage regained in the compressive strength by the water-cured specimen was 9.3% more than that of the air-cured specimen. Similarly, the percentage regained in compressive strengths by the other mixes for the air-cured and water-cured, respectively, were 60.25% and 75.33% for C5, 66.15% and 81.51% for C10, 74.64% and 90.18% for C15, and 79.72% and 92.44% for C20. The strength regained by the water-cured specimen exceeded that of being air-cured by 15.08%, 15.36%, 15.54%, and 12.72% for C5, C10, C15, and C20, respectively. The percentage regained in compressive strength, relative to C0 mix, was 15.02% for C5, 20.92% for C10, 29.41% for C15, and 34.49% for C20 mixes when cured in air and 20.80% for C5, 26.98% for C10, 35.65% for C15, and 37.91% for C20 mixes when cured in water.

The results indicated how compressive strength was regained for all mixes. However, the regain in strength values in the water-cured specimens were higher than those in the air-cured specimens. Furthermore, the regain in compressive strength was observed to increase with an increase in the percentage replacement of MRGC. The regain in compressive strength could indicate that crack-healing activities took place in the specimens. The increase in the strength regain with the content of MRGC could be related to an increase in the availability of unhydrated cement grains, which hydrate to produce new products such as C-S-H and CH that fill and seal the cracks [45,46]. Since MRGC is coarser than OPC, it is more likely that a large portion of the MRGC particles remains unhydrated at the early age of the concrete. Therefore, when cracks form in the concrete and moisture enters through the cracks, the unhydrated MRGC grains and some unhydrated OPC particles may hydrate and heal the cracks. The increase in the strength regain with the increase in the moisture content could be attributed to the presence of enough water for hydration, which could yieldmore hydration products to seal thecracks. Since the crack-healing activity is taking place at the early age of concrete, an argument can arise that the regain in strength could be due to the hydration of the unhydrated OPC particles only. However, the results of the control mix indicated that the hydration of the OPC has a limited effect on healing the cracks when compared to that of MRGC.

#### 3.2.2. Flexural Strength

**Figure 5:**
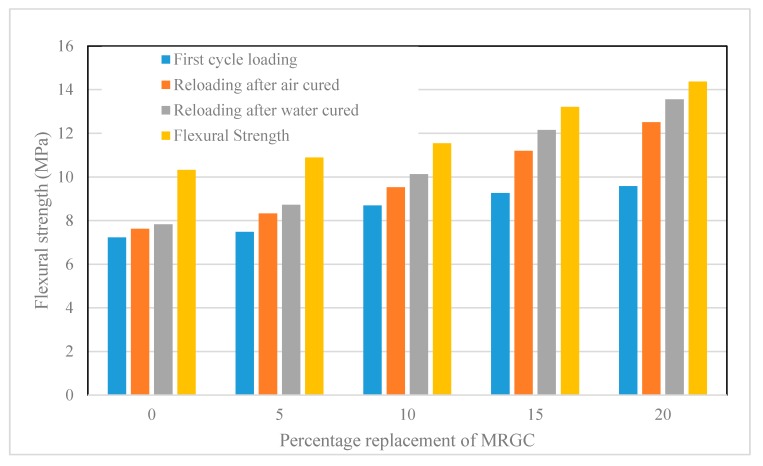
Flexural strength regained due to the crack healing effect of MRGC.

The first cycle flexural strength, the flexural stress required to induce cracks, and the regain in flexural strength due to crack healing are presented in Table 5. A graphical demonstration of the test results is shown in Figure 5.

Similar to the results observed for the compressive strength, the percentage regained in flexural strength of the five mixes, for the air-cured and water-cured, respectively, were 73.84% and 75.87% for C0, 76.49% and 80.07% for C5, 82.58% and 87.78% for C10, 84.78% and 91.98% for C15, and 86.99% and 94.29% for C20. The flexural strength regained by the water-cured specimens exceeded those of air-cured specimens by 2.03%, 3.58%, 5.20%, 7.20%, and 7.30% for C0, C5, C10, C15, and C20, respectively. The percentage regained in flexural strength, relative to the C0 mix, was 2.65% for C5, 8.75% for C10, 10.95% for C15, and 13.15% for C20 mixes when cured in air and 4.20% for C5, 11.91% for C10, 16.10% for C15, and 18.42% for C20 mixes when cured in water.

The results of the flexural strength agree with those of compressive strength such that a general regain in strength was observed in all mixes. However, the regain in strength values of the water-cured specimens were higher than those of the air-cured specimens. The regain in flexural strength also increased with the increase in the content of MRGC. This could be due to the healing of the induced cracks. The increase in the strength regained with the content of MRGC could be related to an increase in the presence of extra unhydrated cement grains, which hydrate and produce additional cement paste responsible for healing the cracks [45,46]. Since MRGC is coarser than OPC, it is more likely that a large portion of the MRGC particles remains unhydrated at the early age of the concrete. Therefore, when cracks form in the concrete and moisture enters through the cracks, the unhydrated MRGC grains and some unhydrated OPC particles may hydrate and heal the cracks. The increase in the strength regain with moisture content could be more water-based for hydration, which could mean more hydration products that could seal more cracks. Since the crack-healing activity is taking place at the early age of concrete, an argument can arise that regaining strength could be due to the hydration of the unhydrated OPC particles only. However, the results of the control mix indicated that the hydration of the OPC has a limited effect on healing the cracks when compared to that of MRGC.

#### 3.2.3. Splitting Tensile Strength Recovery

**Figure 6:**
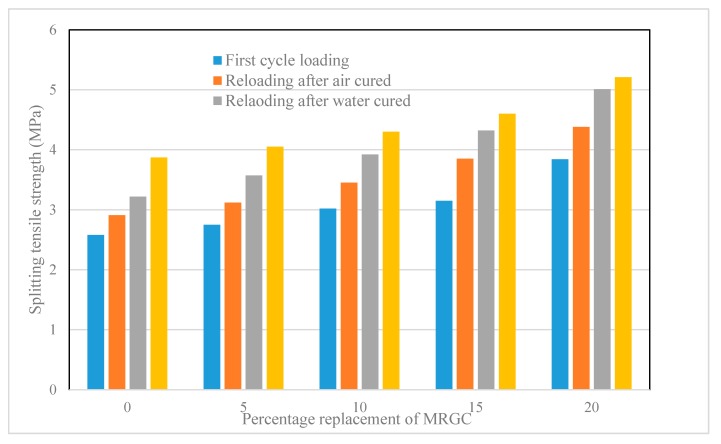
Splitting tensile strength regains due to the crack healing effect of MRGC.

The first cycle splitting tensile strength, the splitting tensile stress required to induce cracks, and the splitting tensile strength regained due to crack healing after the specimens were cured in air and in water are presented in Table 6. A graphical demonstration of the test results is shown in Figure 6.

The trend in the percentage regained in tensile strength was also similar to those of compressive and flexural strengths. The percentage regained in the tensile strength of the five mixes, for the air-cured and water-cured, respectively, were 75.19% and 83.20% for C0, 77.04% and 88.15% for C5, 80.23% and 91.16% for C10, 81.74% and 93.91% for C15, and 84.07% and 96.16% for C20. The tensile strength regained by the water-cured specimens exceeded those of air-cured specimens by 8.01%, 11.11%, 10.93%, 12.17%, and 12.09% for C0, C5, C10, C15, and C20, respectively. The percentage regained in flexural strength, relative to C0 mix, was 1.85% for C5, 5.04% for C10, 6.55% for C15, and 8.88% for C20 mixes when cured in air and 4.94% for C5, 7.96% for C10, 10.71% for C15, and 12.96% for C20 mixes when cured in water.

The results for the tensile strength regained conform with those of flexural and compressive strengths in which a general strength regained was observed in all mixes. However, as in the case of the regain in compressive and flexural strengths, the tensile strength values of the water-cured specimens regained were higher than those of the air-cured specimens. The tensile strength regained also increased with the increase in the content of MRGC, which could be attributed to the crack-healing effect of MRGC due to hydration [45,46].

#### 3.2.4. Water Permeability

**Figure 7:**
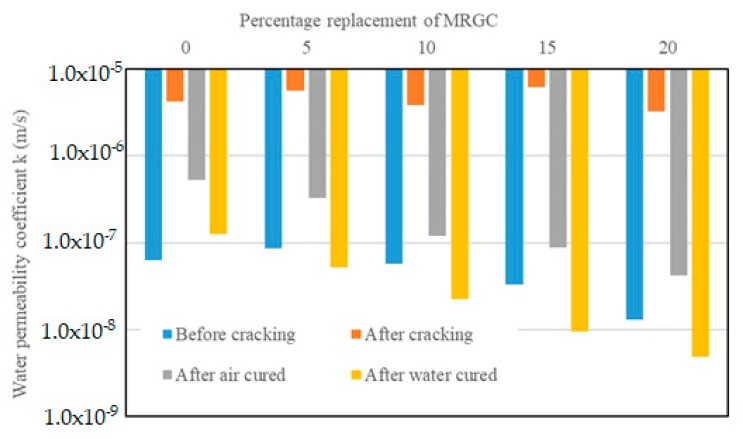
Effect of MRGC content and curing mechanisms on water permeability coefficient k.

The water permeability coefficient k of the concrete specimens before cracking, after cracking, and after the specimens were allowed to heal the induced cracks for 28 days are presented in Table 7. A graphical demonstration of the test results is shown in Figure 7.

The permeability (k values) of the uncracked specimens as a percentage of the permeability of the control C0 were 38.36% for C5, −33.64% for C10, −42.83% for C15, and −59.94% for C20. The drop in the permeability of the air-cured and water-cured specimens, respectively, as a percentage of the permeability of the cracked specimens were 87.67% and 97.06% for C0, 94.24% and 99.08% for C5, 96.86% and 99.41% for C10, 98.61% and 99.85% for C15, and 98.72% and 99.85% for C20.

From the above results, it can be noted that the water permeability of the concrete specimens decreased with the increase of MRGC content for the uncracked specimens and after the crack-healing period. This could be attributed to an increase in the presence of extra unhydrated cement grains [45,46], which hydrate and produce additional cement paste responsible for sealing the cracks, refine capillary pores, and block pore connectivity that would reduce the permeability [46,47,48,49]. The results also indicate that the water permeability coefficient could be affected by the curing mechanism. The specimens cured in water after cracking showed a higher reduction in the values of the coefficient when compared to those cured in air. The water permeability coefficients of the air-cured specimens were higher than those of the reference specimens (specimens without induced cracks) for all concrete mixes. The difference in the values of the coefficient was in the order of magnitude one for C0, C5, and C10. This could be due to the lack of sufficient moisture in the air-cured specimens, which resulted in a limited hydration activity that was essential for healing the cracks. On the contrary, the water permeability coefficients of the water cured specimens were lower than those of the reference specimens for all concrete mixes, except for C0, by a maximum order of a magnitude of one. This indicates that the presence of sufficient moisture helps facilitate the hydration of more unhydrated MRGC grains that could heal the cracks and reduce permeability.

## 4. Conclusions

The following conclusions could be made based on the results from the experimental study on the crack-healing effect of mill-rejected granular cement when used as a partial replacement for a fine aggregate.

- The regain in compressive, flexural, and tensile strengths and the drop in water permeability of concrete after specimens were allowed to heal the induced cracks indicate the occurrence of autogenous crack-healing activities in the concrete.
- The use of mill-rejected granular cement as a partial replacement for a fine aggregate could enable concrete to heal its cracks autogenously. The crack-healing efficiency of concrete could be increased by increasing the MRGC content of the concrete in the presence of sufficient water for hydration.
- The crack-healing effect of MRGC could be attributed to its hydration at a later age. MRGC particles are coarser than OPC particles, which enables the former to hydrate slowly. During the initial hydration, most of the finer OPC particles hydrate while hydration of the MRGC particles is limited to their surface. This could preserve unhydrated cement particles that can be used by concrete to heal cracks forming during its service life autogenously. Therefore, the use of MRGC could help concrete autogenously heal the cracks during its lifetime due to the hydration of the MRGC with the water entering through cracks.
- The mechanical properties of concrete were partially recovered due to the autogenous self-healing capability of concrete with the help of MRGC. The average regained in the mechanical properties of the water-cured specimens was about 90% of the reference strength (first cycle strength).
- The coefficient of permeability of concrete could be reduced by increasing the MRGC content of concrete in the presence of sufficient moisture.
- Based on the trend of the observed results, a further increase in the percentage replacement of MRGC for fine aggregates could improve the mechanical properties and crack-healing efficiency of concrete. However, MRGC is cement by nature and has to be evaluated from an economic viewpoint as well.

## Figures and Tables

**Table 1:** Physical properties and chemical composition of OPC.

**Composition**	**Mass %**
SiO_2_	23.5
Al_2_O_3_	3.5
Fe_2_O_3_	3.2
CaO	63.5
MgO	2.6
Alkalis	0.54
LOI	0.82
Others	2.34
**Physical Properties**	
Specific gravity	3.11
Compressive strength, 28 days (MPa)	30.5
Specific surface, Blaine (m^2^/kg)	390
Initial/final setting (min)	140/480
Normal consistency (%)	30

**Table 2:** Concrete mix proportions (kg/m^3^).

Designation	Cement	Fine Aggregate		Coarse Aggregate	W/C
		Sand	MRGC		
C0	411	838	0	1026	0.47
C5	411	796.1	41.9	1026	0.47
C10	411	754.2	83.8	1026	0.47
C15	411	712.3	125.7	1026	0.47
C20	411	670.4	167.6	1026	0.47

**Table 3:** Concrete slump test results.

Designation	CM0	CM5	CM10	CM15	CM20
Slump (mm)	40	40	35	30	20

**Table 4:** Compressive strength regained due to the crack healing effect of MRGC.

Designation	Average Compressive Strength (MPa) at 28 Days (SD)			
	Strength	Artificial Cracking	Reloading (after Crack-Healing)	
			Air-Cured	Water-Cured
C0	31.52 (0.33)	13.11(0.51)	14.26 (0.34)	17.19 (0.12)
C5	33.13 (0.21)	18.69 (0.32)	19.96 (0.51)	24.96 (0.22)
C10	35.42 (0.5)	22.33 (0.41)	23.43 (0.42)	28.87 (0.34)
C15	36.87 (0.24)	25.31 (0.2)	27.52 (0.4)	33.25 (0.43)
C20	38.21 (0.15)	27.46 (0.16)	30.46 (0.51)	35.32 (0.26)

**Table 5:** Flexural strength regain due to the crack-healing effect of MRGC.

Designation	Average Flexural Strength (MPa) at 28 Days (SD)			
	Strength	Artificial Cracking	Reloading(after Crack-Healing)	
			Air-Cured	Water-Cured
C0	10.32 (0.21)	7.23 (0.5)	7.62 (0.4)	7.83 (0.32)
C5	10.89 (0.14)	7.48 (0.2)	8.33 (0.22)	8.72 (0.33)
C10	11.54 (0.22)	8.69 (0.25)	9.53 (0.30)	10.13 (0.17)
C15	13.21 (0.16)	9.26 (0.18)	11.2 (0.11)	12.15 (0.41)
C20	14.37 (0.20)	9.58 (0.27)	12.5 (0.29)	13.55 (0.50)

**Table 6:** Splitting tensile strength regained due to the crack healing effect of MRGC.

Designation	Average Tensile Strength (MPa) at 28 Days (SD)			
	Strength	Artificial Cracking	Reloading (after Crack-Healing)	
			Air-Cured	Water-Cured
C0	3.87 (0.12)	2.58 (0.15)	2.91 (0.2)	3.22 (0.2)
C5	4.05 (0.11)	2.75 (0.26)	3.12 (0.23)	3.57 (0.13)
C10	4.3 (0.12)	3.02 (0.23)	3.45 (0.3)	3.92 (0.24)
C15	4.6 (0.3)	3.15 (0.34)	3.85 (0.13)	4.32 (0.14)
C20	5.21(0.51)	3.84 (0.33)	4.38 (0.22)	5.01 (0.50)

**Table 7:** Water permeability coefficient k variations due to the crack healing effect of MRGC.

Designation	Coefficient of Permeabilityk (m/s) at 28 Days (SD) (× 10^−7^)			
	Before Cracking	After Cracking	After Crack-Healing	
			Air-Cured	Water-Cured
C0	0.623 (0.08)	42.500 (0.6)	5.240 (0.2)	1.250 (0.1)
C5	0.862 (0.05)	56.400 (1.52)	3.250 (0.12)	0.521 (0.08)
C10	0.572 (0.09)	38.200 (0.56)	1.200 (0.1)	0.226 (0.05)
C15	0.327 (0.09)	62.700 (1.17)	0.870 (0.05)	0.094 (0.01)
C20	0.131 (0.07)	32.900 (1.49)	0.420 (0.07)	0.048 (0.007)
